# Impact of community-delivered SMS alerts on dog-owner participation during a mass rabies vaccination campaign, Haiti 2017^☆^

**DOI:** 10.1016/j.vaccine.2018.03.017

**Published:** 2018-03-23

**Authors:** Julie M. Cleaton, Ryan M. Wallace, Kelly Crowdis, Andy Gibson, Benjamin Monroe, Fleurinord Ludder, Melissa D. Etheart, Marco Antonio Natal Vigilato, Alasdair King

**Affiliations:** a Oak Ridge Institute for Science and Education, 100 ORAU Way, Oak Ridge, TN 37830, United States; b Poxvirus and Rabies Branch, Centers for Disease Control and Prevention, 1600 Clifton Rd, Atlanta, GA 30333, United States; c Christian Veterinary Mission, 19303 Fremont Avenue North, Seattle, WA 98133, United States; d Mission Rabies, 4 Castle Street, Cranborne, Dorset BH21 5PZ, United Kingdom; e Ministry of Agriculture and Natural Resources, Haiti, Boulevard Jean-Jacques Dessalines, Port-au-Prince, Haiti; f Haiti Office, Centers for Disease Control and Prevention, 1600 Clifton Rd, Atlanta, GA 30333, United States; g Pan American Health Organization, 525 Twenty-third Street, N.W, Washington, DC 20037, United States; h Merck Animal Health, 2 Giralda Farms, Madison, NJ 07940, United States

**Keywords:** Rabies, Vaccination campaign, SMS, Text message, mHealth

## Abstract

Haiti has historically vaccinated between 100,000 and 300,000 dogs annually against rabies, however national authorities have not been able to reach and maintain the 70% coverage required to eliminate the canine rabies virus variant. Haiti conducts massive dog vaccination campaigns on an annual basis and utilizes both central point and door-to-door methods. These methods require that dog owners are aware of the dates and locations of the campaign. To improve this awareness among dog owners, 600,000 text messages were sent to phones in two Haitian communes (Gonaives and Saint-Marc) to remind dog owners to attend the campaign. Text messages were delivered on the second day and at the mid-point of the campaign. A post-campaign household survey was conducted to assess dog owner’s perception of the text messages and the impact on their participation in the vaccination campaign. Overall, 147 of 160 (91.9%) text-receiving dog owners indicated the text was helpful, and 162 of 187 (86.6%) responding dog owners said they would like to receive text reminders during future rabies vaccination campaigns. In areas hosting one-day central point campaigns, dog owners who received the text were 2.0 (95% CI 1.1, 3.6) times more likely to have participated in the campaign (73.1% attendance among those who received the text vs 36.4% among those who did not). In areas incorporating door-to-door vaccination over multiple days there was no significant difference in participation between dog owners who did and did not receive a text. Text message reminders were well-received and significantly improved campaign attendance, indicating that short message service (SMS) alerts may be a successful strategy in low resource areas with large free roaming dog populations.

## 1. Introduction

Rabies is a neglected disease that causes human deaths in more than 150 countries worldwide and is primarily spread through the bite of a rabid dog [1]. Those living in poverty and children are over-represented amongst the 59,000 rabies deaths that occur each year [2]. Despite rabies’ notoriety as the deadliest infectious disease in the world, a lack of surveillance and under-utilized or poorly implemented dog vaccination campaigns have hindered global control efforts [3]. Recently local success stories have been reported from programs in Guatemala, Haiti, India and Malawi, with assistance from the Centers for Disease Control and Prevention (CDC), Mission Rabies, and the Pan American Health Organization (PAHO) [4–7]. However, these successes have involved significant staff training and technology incorporation; logistical constraints that cannot be easily replicated in all 122 canine-rabies endemic countries.

While expanding their annual campaigns, Haiti has struggled to improve their vaccination coverages above 45%, and CDC, Christian Veterinary Mission (CVM), Haiti Ministry of Agriculture (MARNDR), Mission Rabies, and PAHO are collaborating to develop novel methods to improve vaccination coverages [7]. Generating sufficient public awareness of vaccination campaigns in low-resource settings can be difficult, as access to TV, radio, and printed media are not routinely available [8]. This is also true in Haiti, where the primary method of vaccination campaign awareness involves megaphone announcements from vehicles several days prior to the campaign, as well as hand-held megaphone announcements by vaccinators on the day of vaccination. In order to overcome this deficiency in public awareness, an option raised in discussion with the stakeholders and Merck Animal Health (known as MSD Animal Health outside of the United States and Canada) was to explore the use of mobile technology.

An estimated 85% of the global adult population owns a cell phone, meaning that in even the least developed countries, cellular data service and familiarity with mobile applications are common-place [9]. An estimated 62% of all Haitians (92% of adults) have mobile phone subscriptions across four major providers [10]. Mobile technology is increasingly evolving to the benefit of public health systems, with recent advances in patient monitoring, health alerts, and disease surveillance [11]. While a review of short message service (SMS) applications in disease prevention noted that very few programs had evaluated the effect of their messages, they have qualitatively reported that beneficiaries found them helpful [12]. mHealth, the use of mobile devices in medicine and public health, has brought recent successes to rabies prevention through vaccination campaign management and bite victim SMS reminders [5,13]. Therefore, in 2017, CDC, CVM, MARNDR, Merck Animal Health, Mission Rabies, and PAHO developed a program to test the impact of a text-based dog vaccination reminder during a mass vaccination campaign in two Haitian communes: Gonaives and Saint-Marc in the Artibonite Department.

## 2. Methodology

The evaluation of the impact of text message reminders on vaccination campaign participation was nested within a larger evaluation of vaccination methodology conducted in Haiti during their 2017 mass dog vaccination campaign. Two vaccination methods were applied in two urban communes (Gonaives and Saint-Marc): a typical campaign that spent 1-day in each vaccination zone in North Saint-Marc and North Gonaives and a mobile application-assisted campaign that spent up to 3 days in each vaccination zone in South Saint-Marc and South Gonaives. The cities were divided along major roadways to help vaccinators find the correct area; there were no significant differences in geological features, population density, or rabies vaccination history. Rabies vaccinators conducted a mixed methodology, in which fixed point vaccination was conducted until participation dropped below 25 dogs per hour, after which vaccination teams switched to door-to-door vaccination. Dog vaccination was conducted 6 days per week over a 17-day period (May 20 – June 5, 2017).

**Fig. 1:**
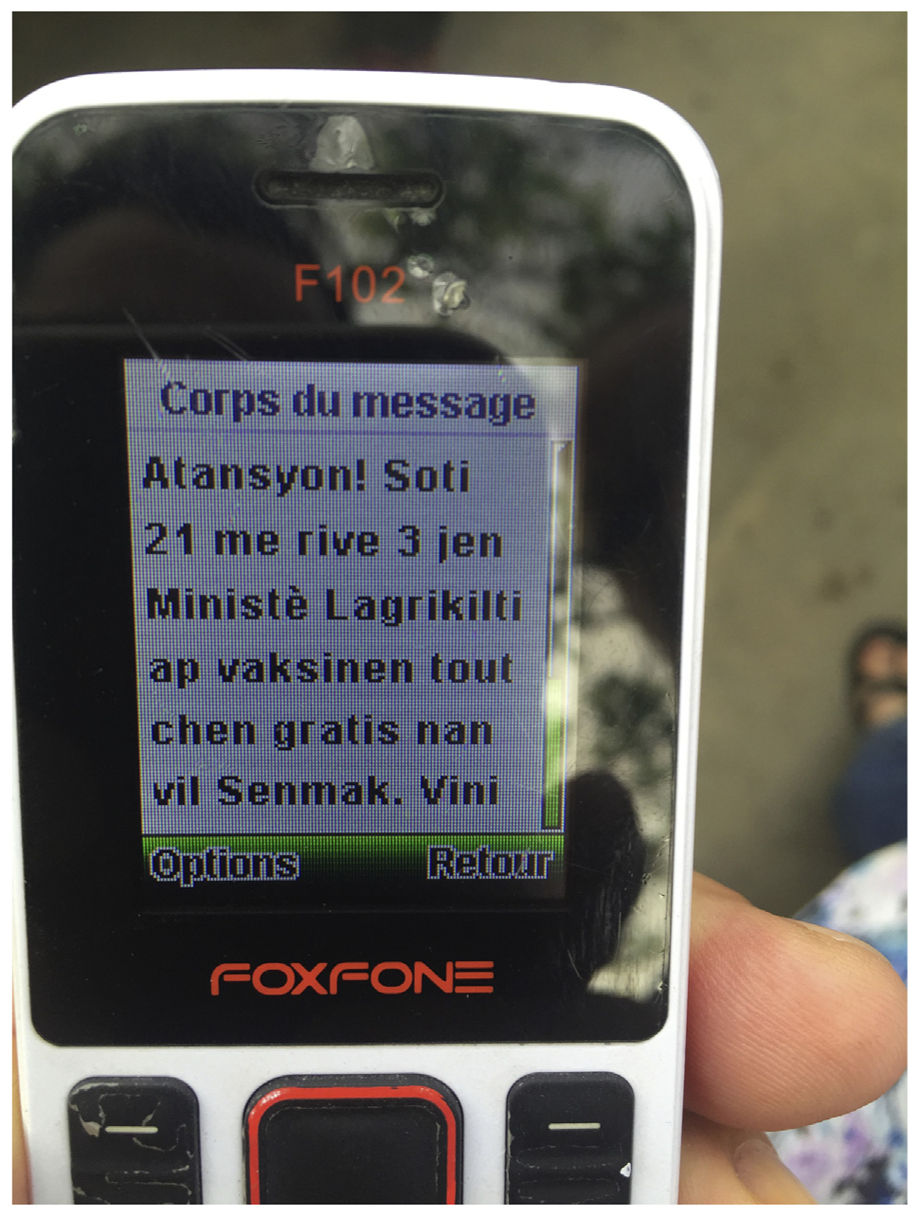
Text message received by community member during Haiti’s mass dog rabies vaccination campaign, 2017. Translates to: “Attention! From May 21 to June 3, the Ministry of Agriculture will vaccinate all dogs in the city of Saint-Marc and Gonaives for free. Take your dogs to vaccinate at the nearest post, to protect yourself and the entire population.”

Merck Animal Health, with assistance from CVM, purchased 600,000 text messages from a major cellular network provider for $10,000 ($0.015 per message). Text messages were delivered to phones with SIM cards purchased in Gonaives and Saint-Marc, regardless of their location within high or low intensity vaccination zones. The text message notified residents of the free rabies vaccination campaign and encouraged them to participate (Fig. 1). On the second day of the campaign, 300,000 texts were delivered. An additional 300,000 were sent at the beginning of the second week of vaccination. Announcers on trucks drove through communities one week before and the night before the campaign. Vaccinators placed wax marks on the forehead of vaccinated dogs, as well as cotton-mesh collars.

Twenty-three of the 231 zones within the communes were randomly selected for post-vaccination coverage assessment, utilizing both household and sight-resight surveys: North Saint-Marc (n = 4), South Saint-Marc (n = 4), North Gonaives (n = 7), South Gonaives (n = 8). The sample size to determine post-vaccination coverage was calculated based on a human population of 292,000, dog ownership rate of 50%, an alpha = 0.05, a design effect of 1.5, and a 10% nonresponse rate. The total number of households to survey was calculated at 634. Interviewers selected a random location within each zone and attempted to interview every other household along a contiguous path until at least 28 households had been interviewed. Surveys were conducted by two surveyors per zone over one to three consecutive days, initiated within 3 days of the vaccination program leaving the area. Sight-resight surveys recorded all dogs seen along paths in each zone, noting the presence of a vaccination mark (wax, collar, or both) to obtain the free-roaming dog vaccination coverage.

To assess campaign awareness and timing of announcement methods, surveyors asked four questions as part of the post-campaign survey (Boxes 1 and 2). A further line of questioning ascertained whether the respondent’s household owned dogs, and if they had brought the dogs to the campaign. The surveyors read the questions and answer options to the interview subjects and recorded their selections. The data were collected in the Mission Rabies application, cleaned in Microsoft Excel^®^, and analyzed in OpenEpi version 3.01 to calculate risk ratios, 95% confidence intervals, and mid-p exact two-tailed probability values.

Box 1 **Survey Questions to Assess Campaign Awareness**

1 ”Did you know that a dog rabies vaccination campaign was taking place in your community?”
  - Yes
  - No
2 ”How did you hear about the campaign?”
  Respondents could select multiple of the following and whether they heard before or after the campaign, or select none if they were unaware:
  - Text message
  - Print media (newspapers, posters, pamphlets)
  - Megaphone
  - Radio
  - Television
  - Friend/neighbor
  - Health care worker
  - Other, specify

Box 2 **Survey Questions to Assess Perception of Text Message Vaccination Announcements**

3 “If you received a text message reminder about the vaccination campaign, was it helpful in your decision to vaccinate your dog?”
  Respondents could select multiple of the following:
  - “The text message I received helped me know when the campaign was in my area”
  - “The text message I received helped me know where the campaign would be held”
  - “The text message I received reminded me to get my dog vaccinated”
  - “The text message I received was not helpful”
  - “I did not get a text message”
4 “Would you like to receive text message reminders about rabies vaccination campaigns in the future?”
  Respondents could select one of the following:
  - “I would like to get text messages about upcoming vaccination campaigns in my area”
  - “I would not like to get text messages, because they are not helpful”
  - “I would not like to get text messages, because I already get too many”
  - “No response”

## 3. Results and discussion

Pooling the four areas of Gonaives and Saint-Marc, 955 household representatives agreed to participate in the survey; 102 declined (participation rate = 90.4%). One zone was interviewed before vaccination and another over a week after; both were removed from the study leaving 682 participants. Dog owners and caretakers composed 33.0% (n = 225) of the survey population, and 160 (71.1%) acknowledged that they had received the SMS. Among those who were aware of the text, 147 of 160 (91.9%) said that it was helpful, primarily to know when the campaign was occurring. The vast majority (n = 162, 86.6%) of responding dog owners said they would like to receive SMS reminders for future campaigns. Only 13.4% (n = 25) of dog owners said they would not like to receive future reminders, split evenly between saying they received too many messages and the content was not helpful.

Text messages were the most frequent method of promoting awareness of the vaccination campaign, with 64% (n = 144) of dog owners responding they were aware by text before the campaign; for 25.8% (n = 58) of respondents, the text message was their only mode of notification. Megaphones were the second most frequently cited awareness method, with 53.3% (n = 120) of dog owners reporting that they were made aware by megaphone, of which 17.3% (n = 39) were notified by megaphone alone. Word of mouth accounted for a further 4.9% (n = 11) of awareness. Only 7.5% (n = 17) of dog owners were unaware of the campaign before it occurred (Table 1).

In the Northern zones, where a less- intensive vaccination effort was applied, text-receivers were significantly more likely to attend the vaccination campaign (73.1% vs 36.4%, p-value = 0.003) (Table 2). The text message was not associated with an increase in vaccination coverage in the Southern zones, where the intensive 3-day campaign was conducted. Among dog owners in both areas who received the text message but no other awareness method before the campaign, 62.5% brought their dogs to be vaccinated, compared to only 12.5% among unaware owners (Rate Ratio: 5, p-value < 0.001). Total estimated free-roaming dog vaccination coverage from the sight-resight post-campaign survey was 43.9% in Northern zones compared to 80.2% in Southern zones. Vaccination coverage among owned dogs was obtained from the household surveys and found 64.0% coverage in Northern zones and 72.8% coverage in Southern zones.

## 4. Conclusion

Reaching 70% rabies vaccination coverage in the dog population is essential to eliminate canine rabies, however achieving these high coverage levels can be difficult in countries with limited resources [14]. Many canine rabies endemic countries must consider vaccination methods that are successful in free-roaming and loosely owned dog populations; these methods are often labor-intensive and costly [15]. Additionally, communications strategies in developing countries can be difficult, and identifying novel methods of awareness and community engagement are needed. This evaluation has shown that, in the context of a traditional 1-day vaccination strategy, text-message campaign reminders may help to significantly improve vaccination coverages in dogs. Megaphone announcements were also an important contributor to campaign awareness and should continue to be used, as they alerted 17% of the population who would not have known otherwise. One limitation of this study is that the network provider services an unknown percentage of the population. Using all providers in the area could have raised awareness by text further.

Over the 2-week vaccination period 11,065 dogs were vaccinated. At a cost of $10,000 for the texting campaign, the text-cost per dog vaccinated was $0.90. Assuming an international average cost per dog vaccinated of $2.18, the cost for the SMS service in this campaign would have increased the cost per dog vaccinated by 41% ($3.08 per dog vaccinated) [16]. If SMS reminders are to be used in future campaigns, organizers should consider cost-sharing, public-private partnerships, or donation services [13,16].

The benefits of text-message reminders were not observed in the 3-day campaign areas; the impact of this awareness method was likely overcome by the labor-intensive door-to-door effort that the vaccination teams conducted. While the text message reminder improved participation in the 1-day vaccination zones, dog vaccination coverage was still below the 70% threshold for rabies elimination. Therefore, increased vaccination intensity in combination with text message reminders may represent a more cost-effective solution to achieving adequate dog rabies vaccination coverage. Future campaigns in Haiti should consider a modified approach of increased vaccination intensity with SMS reminders. This economic relationship should be further explored to maximize the efficiency of dog vaccination programs in countries with limited resources. This evaluation shows that text-message reminders are an effective method to improve community awareness and engagement in mass dog vaccination campaigns in Haiti.

## Figures and Tables

**Table 1:** Frequency of all listed methods of vaccination campaign awareness and combinations thereof; Haiti 2017.

Awareness method	Text message	Megaphone	Friend/neighbor	Radio	Healthcare worker	Television	Print media	Three awareness methods	Unaware	Total (n = 225)
Text message	58 (25.8%)	59 (26.2%)	9 (4%)	6 (2.7%)	1 (0.4%)	0	0	11 (4.9%)	–	144 (64%)
Megaphone	–	39 (17.3%)	8 (3.6%)	2 (0.9%)	1 (0.4%)	1 (0.4%)	0	10 (4.4%)	–	120 (53.3%)
Friend/neighbor	–	–	10 (4.4%)	0	0	0	0	7 (3.1%)	–	34 (15.1%)
Radio	–	–	–	2 (0.9%)	0	0	0	0	–	10 (4.4%)
Healthcare worker	–	–	–	–	1 (0.4%)	0	0	2 (0.9%)	–	5 (2.2%)
Television	–	–	–	–	–	0	0	2 (0.9%)	–	3 (1.3%)
Print media	–	–	–	–	–	–	0	1 (0.4%)	–	1 (0.4%)
Three awareness methods	–	–	–	–	–	–	–	11 (4.9%)	–	11 (4.9%)
Unaware	–	–	–	–	–	–	–	–	17 (7.6%)	17 (7.6%)

* 11 respondents were aware of the campaign through three methods. This column lists the number in each row with any two other awareness methods.

**Table 2:** Campaign attendance by text receipt and location; Haiti 2017.

Gonaives and Saint Marc	Northern Zones			Southern Zones		
	
	Received text	No text	No awareness	Received text	No text	No awareness
Brought dog(s) to campaign	49	8	0	59	32	2
Did not bring dog(s)	18	14	9	26	8	5
% Brought to campaign*	73.1%	36.4%	0.0%	69.4%	80%	28.6%
95% Confidence interval	61.4 – 82.4	19.6– 57.1	0 – 34.5	58.9 – 78.2	65.0 – 89.8	7.6 – 64.8
P-value†	0.003		<0.001	0.22		0.046

* Text receipt was measured here as responding with anything other than “I did not get a text message” to the text helpfulness question and excluding those who answered they received it after the campaign in the awareness question. Lack of awareness was measured as those who did not receive a text or list any other awareness methods they had received before the campaign. Campaign attendance was measured as responding “yes” to “Did you or someone from your household bring your dogs to the vaccination campaign?”.

† Mid-P exact, two-tailed, compared to “Received text” in both cases

## References

[1] Hampson K, Coudeville L, Lembo T, Sambo M, Kieffer A, Attlan M (2015). Estimating the global burden of endemic canine rabies. PLoS Neglect Trop Dis.

[2] World Health Organization (2017). Rabies Fact Sheet [Internet].

[3] Taylor LH, Knopf L (2015). Surveillance of human rabies by national authorities–a global survey. Zoonoses Public Health.

[4] Vigilato MA, Clavijo A, Knobl T, Silva HM, Cosivi O, Schneider MC (2013). Progress towards eliminating canine rabies: policies and perspectives from Latin America and the Caribbean. Philosoph Transact Royal Soc London B: Biolog Sci.

[5] Gibson AD, Ohal P, Shervell K, Handel IG, Bronsvoort BM, Mellanby RJ (2015). Vaccinate-assess-move method of mass canine rabies vaccination utilising mobile technology data collection in Ranchi, India. BMC Infect Dis.

[6] Gibson AD, Handel IG, Shervell K, Roux T, Mayer D, Muyila S (2016). The vaccination of 35,000 dogs in 20 working days using combined static point and door-to-door methods in Blantyre, Malawi. PLoS Neglect Trop Dis.

[7] Wallace R, Etheart M, Ludder F, Augustin P, Fenelon N, Franka R (2017). The health impact of rabies in Haiti and recent developments on the path toward elimination, 2010–2015. Am J Trop Med Hyg.

[8] De Rochars VE, Tipret J, Patrick M, Jacobson L, Barbour KE, Berendes D (2011). Knowledge, attitudes, and practices related to treatment and prevention of cholera, Haiti, 2010. Emerg Infect Dis.

[9] Statista (2017). Number of mobile phone users worldwide from 2013 to 2019 (in billions) [Internet].

[10] World Factbook, U.S. Central Intelligence Agency (2016). Communications: Haiti [Internet].

[11] Gatuha G, Jiang T (2015). KenVACS: Improving vaccination of children through cellular network technology in developing countries. IJIKM.

[12] Déglise C, Suggs LS, Odermatt P (2012). Short message service (SMS) applications for disease prevention in developing countries. J Med Internet Res.

[13] Mtema Z, Changalucha J, Cleaveland S, Elias M, Ferguson HM, Halliday JE (2016). Mobile phones as surveillance tools: implementing and evaluating a large-scale intersectoral surveillance system for rabies in Tanzania. PLoS Med.

[14] Coleman PG, Dye C (1996). Immunization coverage required to prevent outbreaks of dog rabies. Vaccine.

[15] Kaare M, Lembo T, Hampson K, Ernest E, Estes A, Mentzel C (2009). Rabies control in rural Africa: evaluating strategies for effective domestic dog vaccination. Vaccine.

[16] Wallace RM, Undurraga EA, Blanton JD, Cleaton J, Franka R (2017). Elimination of dog-mediated human rabies deaths by 2030: needs assessment and alternatives for progress based on dog vaccination. Frontiers Veterinary Sci.

